# A comparison of optic disc area measured by confocal scanning laser tomography versus Bruch’s membrane opening area measured using optical coherence tomography

**DOI:** 10.1186/s12886-020-01799-x

**Published:** 2021-01-12

**Authors:** Ioana Maria Cazana, Daniel Böhringer, Thomas Reinhard, Charlotte Evers, Diana Engesser, Alexandra Anton, Jan Lübke

**Affiliations:** Eye Center, Medical Center, Faculty of Medicine, University of Freiburg, Killianstrasse 5, 79106 Freiburg, Germany

**Keywords:** Macrodiscs, BMO-OCT, CSLT, Glaucoma

## Abstract

**Background:**

Precise optic disc size measurements based on anatomically exact disc margins are fundamental for a correct assessment of glaucoma suspects. Computerized imaging techniques, such as confocal-scanning-laser-tomography (CSLT), which applies operator defined boundaries and optical-coherence-tomography (OCT), which incorporates an alternative detectable landmark (Bruch’s-membrane-opening (BMO)), have simplified the planimetry of the optic disc and BMO-area, respectively. This study’s objectives are to compare both modalities for area and to define a threshold for macro-BMO using BMO-OCT.

**Methods:**

Retrospectively, patients that simultaneously received CSLT and BMO-OCT scans were included. Their images were correlated and agreement was determined using Bland-Altman-analysis. The diagnostic power of a macro-BMO threshold using OCT was derived after creating a receiver-operating-characteristics-curve using the well-established analogous CSLT threshold (2.43 mm^2^).

**Results:**

Our study included 373 eyes with a median optic disc area by CSLT/ BMO-area by OCT of 2.56 mm^2^ and 2.19 mm^2^ respectively. The Bland-Altman-analysis revealed a systematic deviation with a diverging tendency with increasing area, which enabled the creation of the following mathematical relation: disc-area (CSLT)*0.73 + 0.3 = BMO-area (OCT). BMO-area of 2.19 mm^2^ showed the best diagnostic power for identifying macro-BMOs using OCT (sensitivity: 75%, specificity: 86%).

**Conclusions:**

Area measurements (CSLT optic disc area vs. BMO-area by OCT) showed a systematic deviation with a divergent tendency with increasing size. Our mathematical equation offers an estimated comparison of these anatomically diverse entities. Considering BMO-OCT´ anatomical accuracy, the 2.19 mm^2^ threshold may improve discernment between glaucoma suspects and norm variants.

## Background

Primary open angle glaucoma remains one of the leading causes of blindness worldwide [1]. The condition is defined as a progressive loss of the nerve fiber layer tissue (NFLT) with subsequent visual field damage [2]. It is most commonly clinically detectable as structural alterations to the optic nerve head (ONH) [3]. However, even healthy ONHs display remarkable morphological variations. Additionally, the assessment of the ONH varies greatly depending on examination technique, thus adding to the complexity of the correct diagnosis of glaucoma for even the most experienced physicians [4, 5].

The accurate identification of the optic disc margin, the outer delineation of the NFLT, and the exact calculation of the optic disc size are two key components for all quantitative assessments of the ONH. Nevertheless, great disagreement exists regarding the exact definition of the ONH margin, which in turn can greatly affect the ONH size. While some consider the ring of Elschnig, a dense connective tissue rising up from the anterior segment of the sclera to join Bruch’s membrane and thereby enclosing the choroid, to be the real margin of the ONH [6–8], others regard Bruch’s membrane opening (BMO), which can sometimes extend beyond the border tissue, to be the clinically visible margin as shown in monkeys [9–11]. This increases the complexity of the precise calculation of ONH size. Several measuring techniques (histomorphometry, slit-lamp biomicroscopy, planimetry based on stereophotographs, and computerized imaging), with differing strengths and limitations, have to date been employed. Nevertheless, imaging methods such as confocal-scanning-laser-tomography (CSLT), which applies operator defined boundaries based on the ring of Elsching and optical-coherence-tomography (OCT), which incorporates an alternative detectable landmark (BMO), have automated segmentation and have simplified the planimetry of the area. Moreover, an assessment based on an identifiable landmark, such as the BMO by OCT, could significantly improve clinical evaluations of ambiguous cases. To date, these two modalities have not been directly correlated. Therefore, it is the purpose of our study to put the two imaging modalities (CSLT and BMO-OCT) in direct comparison for area.

Optic disc areas above 2.43 mm^2^ are considered enlarged in CSLT, yet no parallel threshold exists for areas calculated using BMO as a margin by means of OCT. The differentiation between glaucomatous ONHs and macrodiscs, which are large ONHs with a funduscopically thinned NFLT and an enlarged cup, is clinically challenging and can therefore lead to a misdiagnosis. While Jonas et al. (1992) concluded a positive correlation between optic disc size and the NFLT [12], both the Blue Mountain and the Reykavic Eye Study showed a strong correlation between the optic disc size and the susceptibility to glaucoma [13, 14]. These findings reinforce the importance of a prompt and robust detection of macrodiscs. While the BMO-OCT cannot directly discern between macro and non-macrodiscs, its anatomical accuracy in area calculation based on the BMO persuaded us to define a threshold for identifying macro-BMOs.

## Methods

Data acquisition in this single-center retrospective study was performed at the Eye Center, University Hospital Freiburg. The study received approval from the University of Freiburg ethics committee (vote no. 288/19) and followed all tenants of the Declaration of Helsinki. Data acquisition was based on our electronic patient management system. Patients receiving both CSLT and OCT scans without a glaucoma diagnosis from 2014 until the end of 2018 on the same examination day were identified and included in the analysis. CSLT and OCT scans were performed using the Heidelberg Engineering Retina Tomograph III (HRT II/3. ONH Acquisition Module 3.0.7.0; Heidelberg Engineering GmbH, Heidelberg, Germany) and the Spectralis OCT (HRT ONH Viewing Module 3.2.0.0; Heidelberg Engineering GmbH, Heidelberg, Germany). Standard operating procedures were followed. All assessments were carried out by experienced medical technicians.

In CSLT, disc margins were segmented manually by our operators (two in total) and reviewed by a glaucoma expert (three in total). In cases where disc margins showed significant error, images were reassessed by the glaucoma specialist and margins were adjusted. The area was automatically computed using the internal software of the device.

OCT imaging was also carried out by the same medical technicians with the aforementioned commercially available device. Both centration of the scan to the optic disc as well as possible errors in BMO detection were corrected by the same glaucoma specialists. The area within BMO was calculated using the built in software.

Statistical analysis was then performed using R statistical software [15]. A systematic deviation between the two imaging methods was then assessed through a Bland-Altman-plot. On this basis, we created a correlation formula between both methods using linear regression.

Lastly, using the CSLT threshold for macrodiscs (2.43 mm^2^) as a benchmark, we created a receiver operating characteristics-curve (ROC) for the diagnostic power of BMO area as computed through OCT. On this basis we suggested an analogous threshold to detect macro-BMOs using OCT.

## Results

**Fig. 1:**
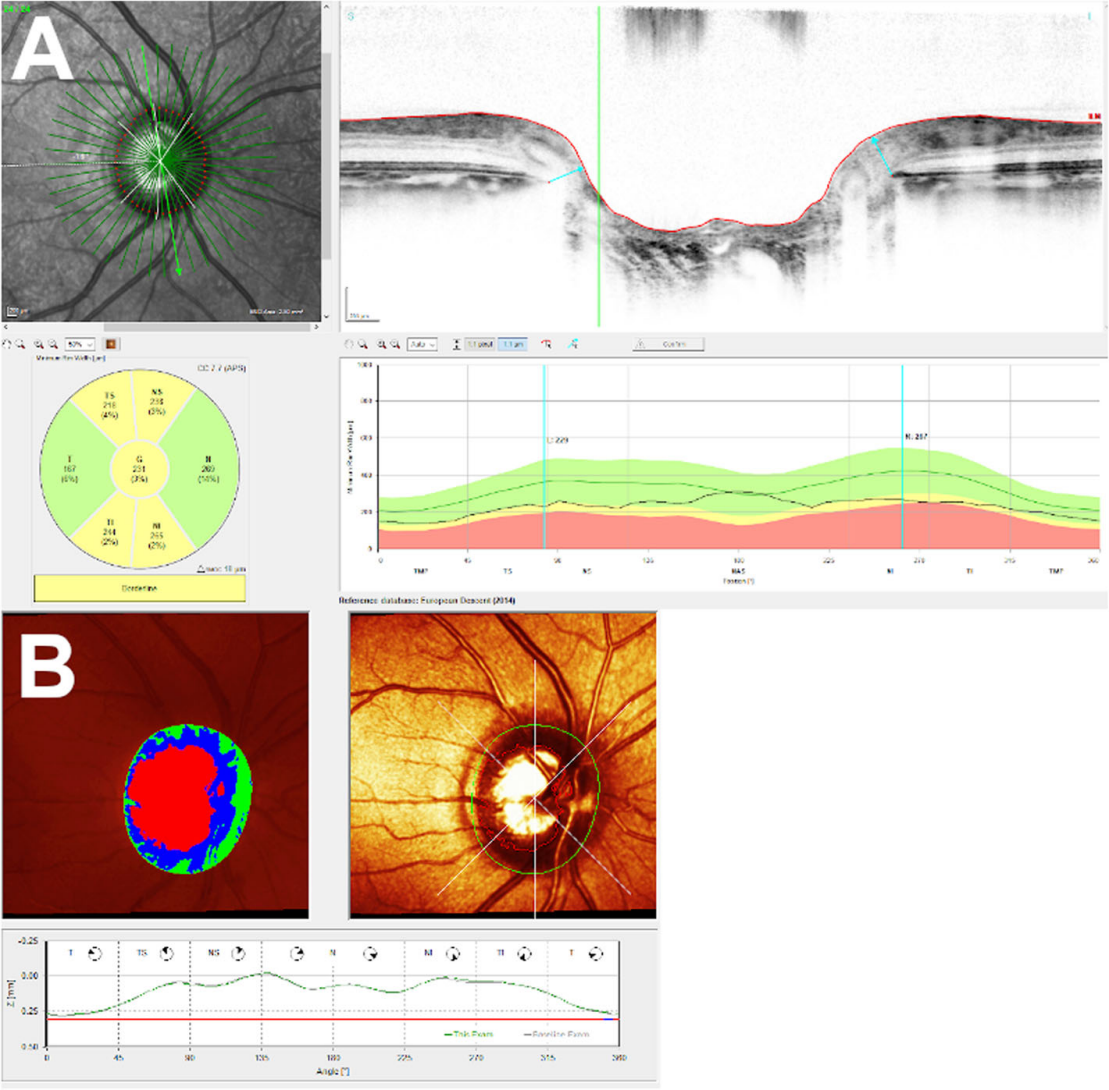
**a** OCT defined BMO- area from a glaucoma suspect’s right eye. **b** CSLT defined optic disc area from the same glaucoma suspect’s right eye

**Fig. 2:**
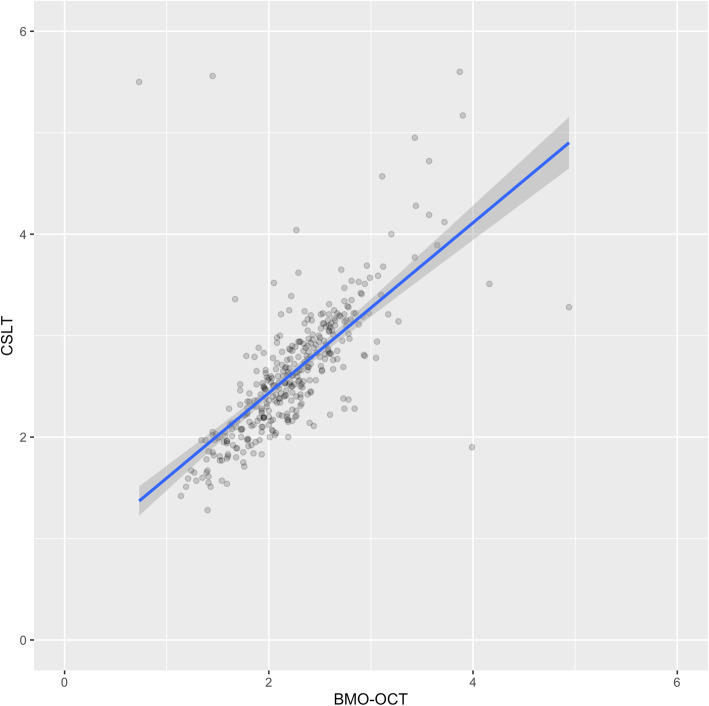
Correlation analysis showing a general good correlation between both area measurement scans (optic disc area by CSLT vs. BMO-area by OCT), with a divergent tendency with increasing size. Darker dots represent multiple data points with the same value. The diagonal blue line represents the line of best fit

373 eyes, with an average age of 52.58 years, were included in our study. Of these, 27.57% had glaucoma, 43.24% remained suspects, and 29.19% showed no signs of the condition. The median visual field mean deviation recorded was 0.4 dB (1st quartile − 0.90 dB, 3rd quartile 2.30 dB). The refractive error was available for 322 eyes and showed a median spherical equivalent of -0.63 D (1st quartile − 2.88 D, 3rd quartile 0.50 D). Table 1 summarizes the aforementioned patient characteristics. The median optic disc area using CSLT was 2.56 mm^2^ (1st quartile 2.19, 3rd quartile 2.95) and the median BMO-area using OCT was 2.19 mm^2^ (1st quartile 1.89, 3rd quartile 2.52). Figure 1 shows a glaucoma suspect’s scans (BMO-OCT 1a and CSLT 1b). The bivariate correlation analysis between the two measurements is shown in Fig. 2. We employed the plot and extrapolated a relation, which was used to create the following equation between the two imaging modalities: Disc Area (CSLT) * 0.73 + 0.3 = BMO-area (OCT).

**Table 1:** Patients characteristics

Number of eyes	373
Mean age	52.58 years
Glaucoma status	
Diagnosed glaucoma	27.57%
Glaucoma suspects	43.24%
Healthy	29.19%
Refractive error (spherical equivalent)	-0.63 D (1st quartile − 2.88 D, 3rd quartile 0.50 D)
Visual field mean deviation	0.4 dB (1st quartile − 0.90 dB, 3rd quartile 2.30 dB)

**Fig. 3:**
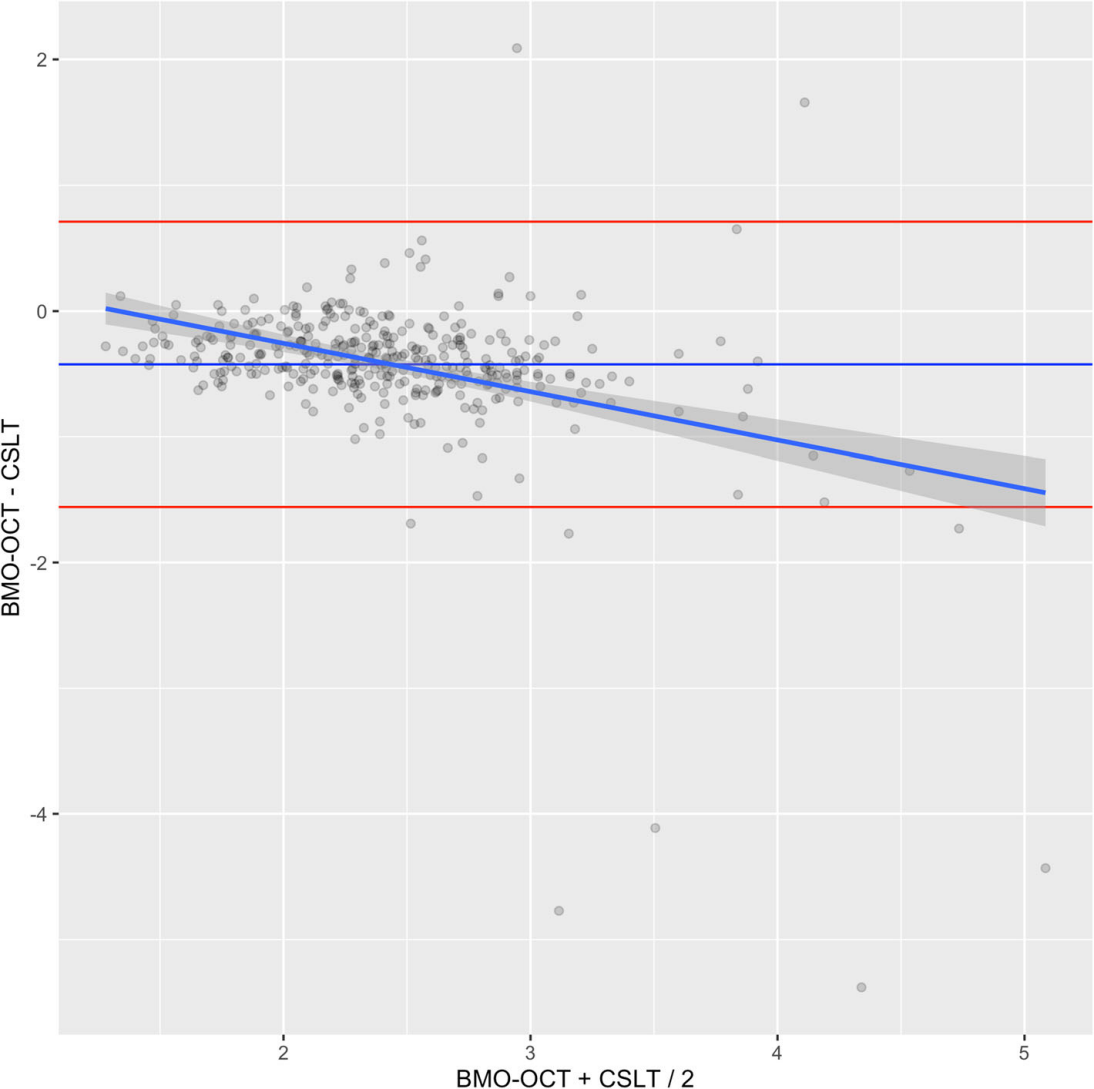
Bland-Altman-Plot representing the difference between both measurement methods for area (optic disc area by CSLT vs. BMO-area by OCT). The red lines represent the standard deviation of the mean difference between the two methods (horizontal blue line) multiplied by 1.96 in both directions. Darker dots represent multiple data points with the same value. The diagonal blue line represents the line of best fit. The mean of the difference between both groups was − 0.42 with a standard deviation of 0.58)

**Fig. 4:**
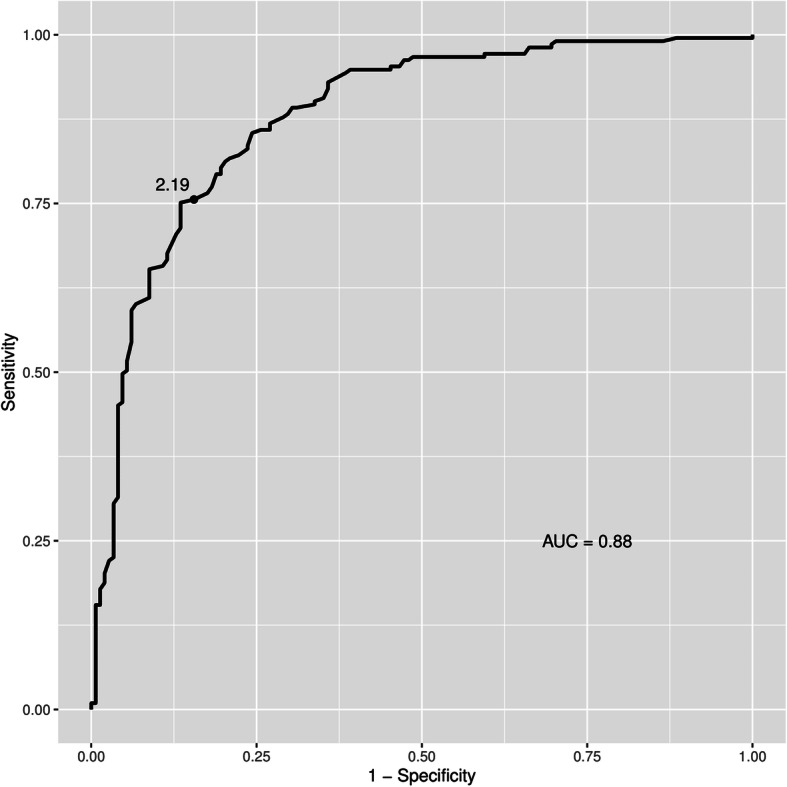
Receiver-operating-characteristics analysis. Discrimination of macro-BMO by OCT

A systematic deviation between the two imaging techniques was noted with a divergent tendency with increasing area as shown in the Bland-Altman-analysis (Fig. 3). The ROC-curve (Fig. 4) showed that a BMO-area of 2.19 mm^2^ or greater in OCT with a 75% sensitivity and 86% specificity had the greatest diagnostic power to discriminate macro-BMOs from norm variants. The area under the curve (AOC) was 0.88.

## Discussion

Our study presents the first comparison between disc area measurements by CSLT versus BMO-area by OCT. We demonstrate a tight correlation between both measuring techniques that weakens slightly with increasing size. On this basis, we suggest a threshold for macro-BMOs using the BMO-area as calculated by OCT (2.19 mm^2^) that parallels the well-established CSLT benchmark (2.43 mm^2^).

It is worth noting that a significant number of our examined ONHs present enlarged discs. At our hospital, patients suspicious of marcodiscs routinely receive both CSLT and BMO-OCT scans. Consequently, this may impact the statistical outcome. Nevertheless, considering the obtained median values for CSLT (2.56 mm^2^) and BMO-OCT (2.19 mm^2^), one notices that mostly borderline ONHs (slightly below or above the macrodisc threshold) were included in our analysis, thus showing clinical relevance.

The optic disc margin is a fundamental landmark as it denotes the outer-most boundary of NFLT of the ONH. Its precise identification is crucial for the correct quantitative assessment of the NFLT. Historically, the ring of Elschnig has accounted for the funduscopically perceived whitish halo representing the ONH margin [8]. This long standing paradigm has been widely challenged following the advent of the OCT, which allows for a 3D insight into the ONH boundary anatomy. Suppot for this theory can be found in a study performed on 28 monkeys which showed that the BMO constituted the disc margin in the majority of eyes [11]. These findings are highly relevant for an accurate interpretation of disc area data. Multiple studies have been performed comparing various disc size measuring techniques. The manual outlining of the ONH boundary based on the perceived ring of Elschnig as required in both stereoscopic photography and CSLT can be highly subjective. Jonas et al. (1998) obtained larger neuroretinal rim area results through CSLT than with disc photography [16]. This was attributed in part to the manual outlining of the margin, which can be better detected in disc photographs versus disc scans [16].On the contrary, OCT tended to measure optic disc size about 10% smaller when compared to disc photographs [17]. Yapp et al. (2018) compared operator-driven (stereoscopic photography and CSLT) and automatic (BMO-OCT) disc size area measuring techniques [18]. The results showed that while CSLT and disc photography showed good correlation, BMO size tended to be smaller when measured by OCT. Additionally, a slightly greater deviation tendency was noted with increasing disc size. These findings are in line with our results, which showed larger disc area measurements by CSLT vs. BMO-areas by OCT, therefore reaffirming the importance of an objective and anatomically precise margin identification process. Our proposed correlation formula may thus provide physicians an anatomically more accurate estimation of the ONH area in centers where only a CSLT is available.

Not only disc margins but also optical magnification can significantly alter disc area results. Littmann (1982) introduced a new formula to determine the true size of the optic disc while taking into consideration both camera and eye magnification [19]. One study looking at highly myopic discs showed better agreement between CSLT and stratus OCT when the OCT results were corrected using the modified axial length method derived from Bennet et al. (1994) [20, 21]. Similarly, Luebke et al. (2017) concluded a mean change of 7.71% in BMO for every 0.3 mm^2^ change in corneal compensation, thus again showing the significance of ocular magnification properties [22]. Our present study did not account for either axial length or corneal refractive errors. Nevertheless, even if corneal compensation could have potentially increased our mean optic disc size the effect should be about the same for both measuring methods.

BMO-based parameters have been analyzed for a number of aberrant disc configurations. The BMO-minimum rim width (MRW) has proven to be superior to other glaucoma detection methods for macrodiscs [23]. In spite of this, CSLT remains a widespread tool even though it has been signaled that the diagnostic value of the Moorfields-Regression-analysis and the Glaucoma Probability Score decreases when analyzing ONHs with extreme sizes. This results in an increase of false positive glaucoma diagnosis for macrodiscs [24]. Since macrodiscs have a misleading appearance which can often lead to a misdiagnosis of glaucoma, the correct identification and assessment of this entity via instrument based diagnostic becomes vital. When looking at macrodiscs (disc area > 2.43 mm^2^ in CSLT), one study obtained larger mean disc areas in glaucomatous, non-glaucomatous and ocular hypertension patients through BMO-OCT versus CSLT [23]. This may in part be due to the systematic deviation between the two imaging techniques with increasing disc area we have observed. Additionally, macrodiscs tend to be underexposed because of the intense reflection of the large cup area when brightness control is left in the automatic mode in CSLT, therefore complicating defining the exact margin [25]. This argument reinforces the relevance of an automatic and accurate identification of the BMO-margin as enabled by OCT.

OCT has become readily available to many physicians and the BMO-analysis is shown to have a superior diagnostic power to all other parameters for macrodiscs [23]. These factors along with the argument that glaucoma susceptibility increases with increasing disc size require physicians to correctly identify patients. Our proposed threshold for macro-BMO, computed by means of OCT (2.19 mm^2^), simplifies and automates the detection process of discs at risk.

## Conclusions

Computerized imaging techniques have significantly enhanced the glaucoma detection process and consequently the evaluation of its progression. With its three-dimensional insight into the ONH structures and its objectivity, the BMO-OCT allows for a precise quantitative assessment of the NFLT for a variety of aberrant ONHs. Therefore, our proposed threshold for macro-BMOs as computed through OCT (2.19 mm^2^), with a 75% sensitivity and an 86% specificity, simplifies and automates the detection process and enables an anatomically correct identification of macro-BMO. Our data comparing optic disc area measurements by CSLT to BMO-area measurements by OCT show a good correlation with a systematic deviation and a divergent tendency with increasing disc size. This reinforces the importance of an accurate insight into border anatomy for aberrant discs, such as the macrodiscs, in order to correctly discern between glaucoma suspects and norm variants.

## Data Availability

The datasets used and/or analyzed during the current study are available from the corresponding author on reasonable request.

## Abbreviations

BMO: Bruch’s membrane opening
OCT: Optical coherence tomography
BMO-OCT: Bruch’s membrane opening optical coherence tomography
CSLT: Confocal scanning laser tomography
ONH: Optic nerve head
NFLT: Nerve fiber layer tissue
